# COVID-19: A Possible Cause of Spontaneous Pneumoperitoneum

**DOI:** 10.2478/jccm-2023-0018

**Published:** 2023-07-31

**Authors:** Patrícia Varela Ramos, Ana Maria Oliveira, Ângela Simas, Margarida Rocha Vera Cruz

**Affiliations:** Intensive Care Unit, Hospital de Vila Franca de Xira, Vila Franca de Xira, Portugal

**Keywords:** COVID-19, pneumomediastinum, pneumoperitoneum, subcutaneous emphysema, Macklin effect

## Abstract

**Introduction:**

Pneumoperitoneum is the presence of air within the peritoneal cavity and is mostly caused by organ rupture. Spontaneous pneumoperitoneum accounts 5% to 15% of the cases and occurs in the absence of organ damage. The pulmonary origin of pneumoperitoneum is unusual, and probably associated with mechanical ventilation and alveolar leak. In patients with coronavirus disease 2019 (COVID-19) there are some reports of air leak, like pneumothorax, pneumomediastinum, pneumoperitoneum, and subcutaneous emphysema.

**Case presentation:**

We present the case of a 70-year-old man with COVID-19 pneumonia admitted to the Intensive Care Unit (ICU). Since admission he was on Non-Invasive Ventilation (NIV), without improvement, needing Invasive Mechanical Ventilation (IMV) due to severe respiratory failure. Five days after IMV despite protective lung ventilation, massive spontaneous subcutaneous emphysema, pneumomediastinum and pneumoperitoneum were diagnosed. Besides initial conservative management 12 hours later, the patient developed abdominal compartment syndrome requiring percutaneous needle decompression.

**Conclusions:**

Pneumoperitoneum can be considered a rare complication of COVID-19 pneumonia and its management, resulting not only from the viral pulmonary but also from secondary causes. Conservative management should be usually enough. However, in the presence of abdominal compartment syndrome prompt recognition and treatment are crucial and eventually lifesaving.

## Introduction

Pneumoperitoneum is the presence of air collection in the peritoneal cavity. In 85–95% of cases, pneumoperitoneum is secondary to visceral perforation, for that requires frequently urgent abdominal surgery. However, pneumoperitoneum may develop spontaneously (5–15%) and in this scenario surgical management is unneeded [1, 2]. Spontaneous pneumoperitoneum (SP) has been associated with intrathoracic, intra-abdominal and gynecological diseases, as well as iatrogenic causes. Thoracic causes of SP include chest trauma, cardiopulmonary resuscitation, tracheal rupture, Ventilator induced lung injury (VILI), pneumothorax and pneumomediastinum. VILI and lung frailty predispose to air leak due to subpleural alveolar rupture. The free air released dissects the peribronchovascular sheath and reaches the mediastinum, pleural cavity, and subcutaneous tissues, through the so-called Macklin effect. Thoracic free air passes the diaphragm to abdominal cavity, leading to SP [1, 3].

There are several case reports of air leak in patients with COVID-19 pneumonia, either spontaneous or mechanical ventilation-related, regarding the possible direct effect of the virus on the lung [1, 3]. Mostly air leaks were pneumothorax (68%), pneumomediastinum (47%), and subcutaneous emphysema (34%) [4]. Spontaneous pneumoperitoneum associated to COVID-19 pneumonia is rare, with few reports in the literature [2, 5]. Usually, pneumomediastinum, subcutaneous emphysema and pneumoperitoneum are managed conservatively, while pneumothorax commonly needs chest drain insertion. In COVID-19 patients, air leak development was associated with a higher need for IMV, longer ICU length of stay (LOS), and higher mortality [6, 7].

We present a case of spontaneous pneumoperitoneum in a patient with severe COVID-19 pneumonia that underwent IMV.

## Case presentation

A 70-year-old man presented to the Emergency Department (ED) with 4 days of increasing shortness of breath and fatigue, having a positive SARS-CoV-2 screening test 15 days before. His past medical history included arterial hypertension, rheumatoid arthritis and prostate cancer under radiotherapy and hormone therapy.

At ED, he denied fever, chills, chest and abdominal pain, nausea, or vomiting. He had no fever, but was breathless, tachypneic, and severely hypoxemic with a peripheral oxygen saturation of 64% under room air, improving to 98% on 15L of oxygen/minute via a nonrebreather mask. Chest examination revealed bilateral crackles and wheezing. Arterial blood gas analysis showed a respiratory alkalosis and hypoxemia (arterial oxygen partial pressure - PaO_2_/fractional inspired oxygen - FiO_2_ ratio 170). Laboratory tests showed an elevated C-reactive protein (43.73 mg/dL) and D-dimer (35 200 ng/mL) and acute kidney injury AKIN 2. The chest X-ray revealed bilateral reticulonodular infiltrates and linear opacities with a predominantly peripheral distribution. COVID-19 pneumonia was diagnosed. He was admitted to the general ward and dexamethasone was promptly started maintaining oxygen via non-rebreather mask, therapeutic-dose anticoagulation, and diuretic therapy. In the first 48h he developed severe respiratory failure and was transferred to the ICU, requiring IMV, after a failed trial of NIV with progressive ascent parameters until Continuous positive airway pressure 12 cmH_2_O and FiO_2_ 90%, for about 24 hours.

**Fig. 1.**
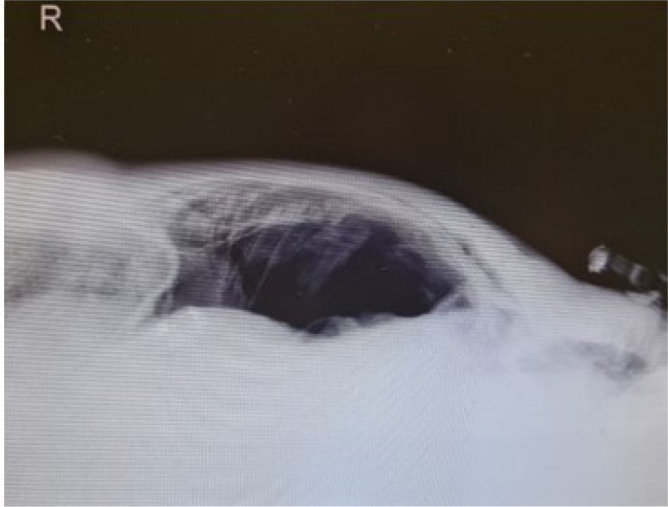
Tangencial X-ray of abdominal showing free gas in abdominal cavity.

**Fig. 2.**
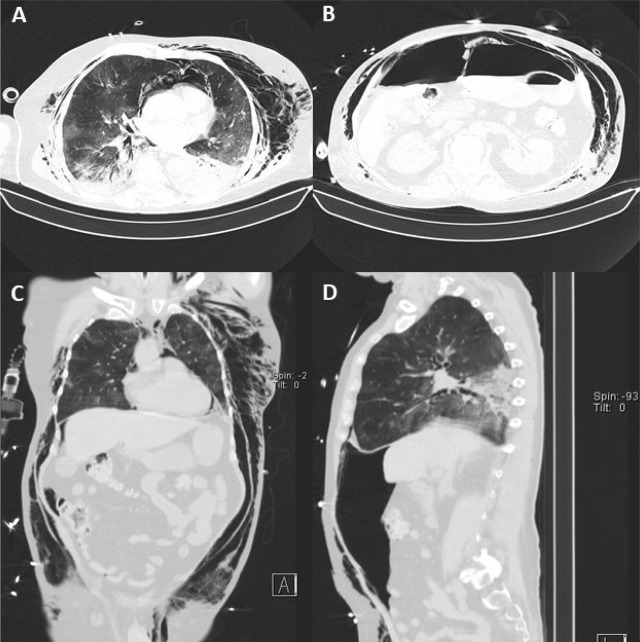
Thoraco-abdominal CT showing SE and moderate pneumomediastinum (axial - A, coronal - C) and a large pneumoperitoneum (axial - B, sagital - D).

**Fig. 3.**
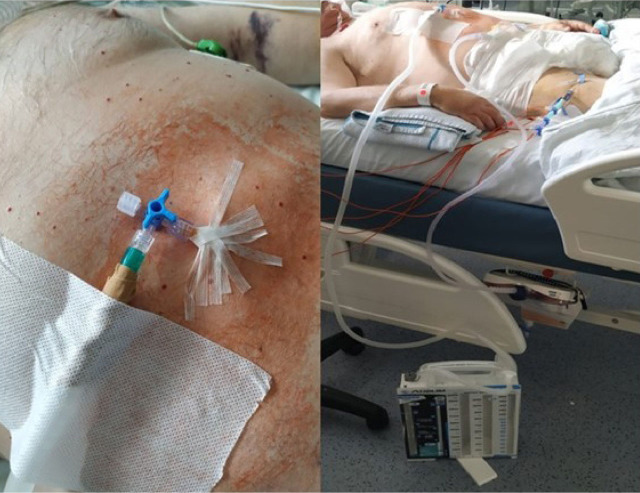
Emergency percutaneous needle decompression. The catheter was connected to an underwater seal drainage system.

The patient remained deeply sedated on pressure-controlled mode ventilation. Due severe respiratory failure, neuromuscular blocking agents were applied during the first 72h, and then by short intermittent infusion or bolus as needed basis to reduce patient-ventilator asynchrony and to access respiratory mechanics. Recruitment manoeuvres were applied once by stepwise approach reaching a peak inspiratory pressure of 35 cmH_2_O and respecting 15 cmH_2_O of driving pressure. Then the PEEP was set for the best compliance and low driving pressure. Oxygenation and pulmonary compliance improved without haemodynamic instability. Lung-protective ventilation was maintained with low tidal volume around 6 mL/Kg of predicted body weight, plateau pressure below 30 cmH_2_O, PEEP of 15 cmH_2_O and FiO_2_ of 55%. Dynamic lung compliance was 30 mL/cmH_2_O and PaO_2_/FiO_2_ ratio stayed above 150, with no need of prone positioning. On 5^th^ day of IMV, cervical, thoracic and abdominal subcutaneous emphysema were noted on physical exam and pneumomediastinum was suspected on chest X-ray. Abdominal distension and tympanic percussion were also observed, with tangential X-ray of abdomen showing free gas in abdominal cavity (Figure 1). A thoraco-abdominal computed tomography (CT) scan was performed and confirmed subcutaneous emphysema, moderate pneumomediastinum and a large pneumoperitoneum, without pneumothorax, tracheoesophageal rupture, or signs of visceral perforation (Figure 2). In the absence of bowel perforation and other clinical consequences, the multidisciplinary team (intensivists and general surgeons) decided to manage conservatively the SP. However, 12 hours later, the patient became unstable. Lung mechanics were changed (tidal volumes and lung compliance decreased, peak inspiratory and mean airway pressures increased), hypoxemia worsened and intra-abdominal pressure (IAP) increased without signs of pneumothorax. An abdominal compartment syndrome (IAP 25 mmHg and respiratory disfunction) developed, and emergency percutaneous needle abdominal decompression with a 14-G venous catheter was performed (Figure 3). The catheter was connected to an underwater seal drainage system (Figure 3), with clinical improvement allowing catheter removal after three days, with complete resolution and no relapse of the pneumoperitoneum. He did not present any features suggestive of gastrointestinal tract perforation (nausea, vomiting, diarrhoea, abdominal pain, or hemodynamic instability). Thereafter, abdominal examination remained normal, enteral feeding was fully tolerated and bowel function remained normal.

Due to prolonged mechanical ventilation, critical illness polyneuropathy and delirium, a percutaneous tracheostomy was performed on day 22 of IMV. The patient was discharged to the ward 48 days after ICU admission and left the hospital on day 129.

## Discussion

We present a rare case of spontaneous pneumoperitoneum (without visceral perforation) in a COVID-19 patient successfully managed by minimally invasive technique instead of an unnecessary laparotomy [5]. The thoracic origin of pneumoperitoneum is mostly associated with mechanical ventilation, especially with high-pressure management [2].

In the present case, viral infection was diagnosed 2 weeks before and signs of respiratory distress began 4 days before admission. In fact, this disease is manifested by a silent hypoxaemia with critically low PaO_2_ associated with a disproportionate mild respiratory discomfort and dyspnoea. Spontaneous breathing during this period, and also the NIV trial, in an already damaged lung, could lead to an excessive respiratory drive and effort and contribute to patient-self-inflicted lung injury (P-SILI). In this case, patients’ lungs under NIV could have been more exposed to the high pressures of mechanical ventilation, a factor promoting lung injury. Monitoring transpulmonary driving pressure, through esophageal balloon, and electrical impedance tomography can be additional tools to ensure a protective ventilation and improve patient outcomes, particularly in intra-abdominal hypertension situations [8].

In the subset of patients with COVID-19, it seems that the development of air leak cannot be explained by pulmonary barotrauma alone. Lungs are inflamed, alveoli are de-recruited, airways are swollen, with consequent inhomogeneous lung ventilation and consequently low compliance and elastance [3, 9]. In the fibrotic and hypoelastic lung, there could be a pressure gradient between the alveoli and the pulmonary interstitium responsible for the generation of high transalveolar pressures, which makes the lung units more susceptible to alveolar rupture and subsequent air leak into the interstitium. Valsalva manoeuvres, cough, and increased work of breathing can also contribute to high transalveolar pressures [2, 3, 5, 9, 10]. According to Lemmers et al., the average day of appearance of pneumomediastinum/subcutaneous emphysema is 3.5 days (SD 0.25–7.5) following intubation, under “protective” ventilatory strategy. Interestingly, when pneumomediastinum was detected, airway pressures were lower compared to the time at which mechanical ventilation was started [11].

Although VILI is considered the most common thoracic cause of pneumoperitoneum, a rising trend in the setting of COVID-19 patients who do not initiate IMV suggests a possible direct effect of the virus [2]. In fact, pneumomediastinum occurred at a moment when airways pressures were not elevated, corroborating that its aetiology can be explained more by “lung frailty” caused by the underlying disease process of COVID-19 than by mechanical ventilation [3, 11].

In the present case, the aetiology of pneumoperitoneum could be explained mainly by the combination of 2 factors: lung frailty induced by SARS-CoV-2 and VILI. Despite protective ventilation, lung recruitment manoeuvres (LRM) were performed. LRM can reduce atelectasis and reduce intrapulmonary shunt as well as increase end-expiratory lung volume and pulmonary compliance. Despite this benefit, they may be associated with complications, including hemodynamic compromise and barotrauma. Usually, they are applied if hypoxemia is refractory (PaO_2_/FiO_2_ <100) despite optimization of therapy, in the absence of contraindication, but its application in ARDS remains controversial [12]. This transient and sustained increase in transpulmonary pressure can cause overdistension of alveoli in well-ventilated lung areas, marked increase in ventilation-perfusion mismatch, barotrauma, and pneumothorax. The absence of pneumothorax, despite the presence of pneumomediastinum, is also interesting in our case. The air can reach the mediastinum without causing a significant damage in the alveolar lung parenchyma [3]. Some authors believe that it can be explained by reactive pleural thickening caused by SARS-CoV-2 that prevents the spreading of the air to the pleural space [9].

We also wonder about the role of tracheal intubation in its pathophysiology. We know that COVID-19 causes central and upper airway inflammation and oedema which make patients more prone to injury resulting from instrumentation. Unquestionably intubation on emergent setting can potentiate the tracheobronchial injury. Despite CT scan ruled out tracheal lacerations, a small upper airway injury might have been undetected.

Undoubtedly an emergency intubation, IMV, a high PEEP and recruitment manoeuvres are all factors that contribute to an increase in the incidence of pneumomediastinum, subcutaneous emphysema and pneumoperitoneum. Nevertheless, according to the literature, “lung frailty” caused by SARS-CoV-2 has a particular importance in its aetiology and must be considered.

Usually, spontaneous pneumomediastinum can be conservatively managed because unnecessary surgical interventions can be harmful in critically ill patients with low PaO_2_/FiO_2_ ratio. In the absence of tract gastrointestinal perforation, pneumoperitoneum is usually self-limited. If abdominal compartment syndrome developed, emergent needle decompression can be needed. Prompt recognition and treatment are essential [5].

## Conclusion

Spontaneous pneumomediastinum is a rare complication of COVID-19 pneumonia and its management, resulting from viral pulmonary lesion or secondary to airway trauma during tracheal intubation, VILI or recruitment manoeuvres [2, 5, 9]. Pulmonary origin of pneumoperitoneum is unusual, and it is caused by air leak with subsequent dissection into the abdominal compartment.

Although the precise mechanism of pneumoperitoneum in these patients is debatable, several mechanisms could be implicated. Future research must be carried out to clarify its pathophysiology.
